# Nitrogen Fixation in Thermophilic Chemosynthetic Microbial Communities Depending on Hydrogen, Sulfate, and Carbon Dioxide

**DOI:** 10.1264/jsme2.ME17134

**Published:** 2018-03-29

**Authors:** Arisa Nishihara, Shin Haruta, Shawn E. McGlynn, Vera Thiel, Katsumi Matsuura

**Affiliations:** 1 Department of Biological Sciences, Tokyo Metropolitan University Minami-Osawa, Hachioji, Tokyo 192–0397 Japan; 2 Earth-Life Science Institute, Tokyo Institute of Technology Ookayama, Meguro-ku, Tokyo 152–8551 Japan; 3 Biofunctional Catalyst Research Team, RIKEN Center for Sustainable Resource Science Wako-shi 351–0198 Japan; 4 Blue Marble Space Institute of Science Seattle, WA 98145–1561 USA

**Keywords:** nitrogen fixation, chemoautotrophic bacteria, thermophilic bacteria, microbial mats, sulfate reduction

## Abstract

The activity of nitrogen fixation measured by acetylene reduction was examined in chemosynthetic microbial mats at 72–75°C in slightly-alkaline sulfidic hot springs in Nakabusa, Japan. Nitrogenase activity markedly varied from sampling to sampling. Nitrogenase activity did not correlate with methane production, but was detected in samples showing methane production levels less than the maximum amount, indicating a possible redox dependency of nitrogenase activity. Nitrogenase activity was not affected by 2-bromo-ethane sulfonate, an inhibitor of methanogenesis. However, it was inhibited by the addition of molybdate, an inhibitor of sulfate reduction and sulfur disproportionation, suggesting the involvement of sulfate-reducing or sulfur-disproportionating organisms. Nitrogenase activity was affected by different O_2_ concentrations in the gas phase, again supporting the hypothesis of a redox potential dependency, and was decreased by the dispersion of mats with a homogenizer. The loss of activity that occurred from dispersion was partially recovered by the addition of H_2_, sulfate, and carbon dioxide. These results suggested that the observed activity of nitrogen fixation was related to chemoautotrophic sulfate reducers, and fixation may be active in a limited range of ambient redox potential. Since thermophilic chemosynthetic communities may resemble ancient microbial communities before the appearance of photosynthesis, the present results may be useful when considering the ancient nitrogen cycle on earth.

N_2_-fixing microorganisms (diazotrophs) are commonly distributed in various habitats in which the lack of bioavailable nitrogen limits primary production. Biological nitrogen fixation is often associated with carbon fixation or autotrophy (5, 10, 17, 26), and previous studies have focused on photosynthetic organisms as the primary producers. In the ocean, free-living or symbiotic cyanobacteria have been identified as key players in nitrogen input (77). In terrestrial ecosystems, several lineages of heterotrophic N_2_-fixing bacteria associate with higher plants, such as rhizobial and actinorhizal symbioses, or endophytes in legumes and monocots (including rice, maize, or wheat), supplying fixed nitrogen in return for fixed carbon from host plants (30, 36, 57, 63, 65).

Chemoautotrophs use reduced sulfur, nitrogen, iron species, or hydrogen to fix inorganic carbon by gaining energy from the oxidation of reduced compounds. These organisms are found in a number of habitats, including marine hydrothermal vents and methane seeps as well as terrestrial caves and hot springs (13, 14, 33, 35). In these habitats, fixed carbon is used by these organisms for growth and also to support other organisms in the ecosystem (6, 33).

Molecular-based studies targeting the *nifH* gene, encoding for the nitrogenase reductase subunit of nitrogenase, the key enzyme in nitrogen fixation, have suggested the distribution of potential diazotrophs in various chemoautotrophic ecosystems. For example, the presence of the *nifH* gene and nitrogenase activity have been reported in chemoautotrophic symbionts associated with lucinids (39, 60) in shallow-water coastal sediments and amphipods in terrestrial caves (13), deep-sea methane seep sediments (12, 48), and hydrothermal vents (7, 45, 54). In terrestrial hot springs, nitrogenase activities *in situ* and in enrichment cultures, as well as *nifH* gene transcription, were detected in sediments of hot springs at 76–86°C in Yellowstone National Park (20, 21, 42). However, the biological characterization of nitrogen fixation in terrestrial thermophilic chemoautotrophic communities has not yet been reported.

In terrestrial hot springs, chemoautotrophic bacteria and archaea are the primary producers in a temperature range higher than 70°C, whereas anoxygenic and oxygenic photosynthetic bacteria are major producers at temperatures less than 70°C in most neutral and alkaline hot springs (3, 15, 47). This temperature dependence of the distribution of primary producers may correspond to the early evolution of energy-transducing systems because highly thermophilic chemoautotrophs are widely distributed in deep branches of the evolutionary tree of life, whereas less-thermophilic photosynthetic organisms are distributed in relatively-recent branches in the tree (37, 68). Therefore, a deeper understanding of the distribution of nitrogen fixation in thermophilic chemosynthetic organisms is important for elucidating the early evolution of this metabolism.

Nakabusa hot springs (Nagano, Japan) is a well-studied terrestrial thermophilic environment (15, 23, 27, 33, 34, 40, 52, 53, 56, 58, 71, 74, 75). These hot springs are sulfidic and slightly alkaline, with a maximum water temperature >90°C. In Nakabusa, several types of dense microbial communities, so-called steamers (filamentous microbial communities) or microbial mats (layered microbial communities), develop in streams and on the surface of a concrete sediment-control dam wall, which is partially covered with hot spring water, with temperatures ranging between 45°C and 80°C. Microbial community compositions in the spring were initially examined using denaturing gradient gel electrophoresis (DGGE) targeting 16S rRNA genes by Nakagawa and Fukui (52). The anoxygenic phototrophic filamentous bacteria, *Roseiflexus castenholzii* (24) and *Chloroflexus aggregans* (23) belonging to the phylum *Chloroflexi* have been isolated from microbial mats. Community diversification at various temperatures in differently colored microbial mats was previously reported in detail (15). Interspecies interactions have been studied in anoxygenic phototroph-dominated mats, with a focus on sulfide, hydrogen, and carbon consumption and production (40, 50, 58).

Chemoautotrophic microorganisms dominate the microbial communities found at >70°C in these hot springs, in which photosynthesis is not possible. Various colors (colorless, white, gray, pink, black, and others) of streamers and microbial mats (or biofilms) are often observed in various hot spring ecosystems around the world (USA [Yellowstone National Park], Iceland, Russia [Kamchatka], Thailand, China, Portugal, and Japan) (11, 28, 46, 47, 52, 61, 64, 72, 76). These communities, similar to the mats and streamers in Nakabusa (52), typically contain microbes of the phylum *Aquificae*, a thermophilic bacterial lineage with chemotrophic, H_2_, sulfide, elemental sulfur, and/or thiosulfate oxidizing members (29), and possibly ancient relict anaerobic metabolism (18). Other typical members of the communities are *Thermus-Deinococcus*, *Thermotogae*, *Thermodesulfobacteria*, *Firmicutes*, uncultured *Crenarchaeota*, and methanogenic *Euryarchaeota* (see the above references).

Nitrogen metabolism in these chemoautotrophic communities has not yet been elucidated in detail. In order to clarify the potential of nitrogenase activity in the communities, we examined nitrogenase activity in the microbial mats that developed at temperatures higher than 70°C in the hot spring water of Nakabusa, Japan. We employed inhibitor-supplement experiments to characterize the factors affecting nitrogenase activity and suggest the involvement of chemosynthetic metabolism, using hydrogen, sulfate, and carbon dioxide, for the nitrogen fixation.

## Materials and Methods

### Sample collection

**Fig. 1:**
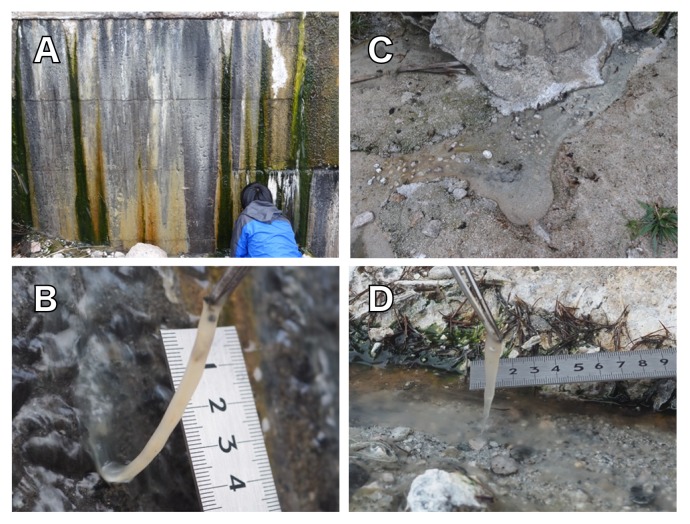
Photo images of two sampling sites (A, C) and typical pale tan colored microbial mats (B) and streamers (D) at Nakabusa hot springs. **A**, Microbial mats developed on a concrete wall that hot spring water runs down (Wall Site); **B**, typical pale tan colored microbial mats at 72–75°C at the Wall Site; **C**, a hot spring water stream in which pale tan colored streamers are observed (Stream Site); **D**, typical pale tan colored streamers at 75°C at the Stream Site.

Samples were collected from two hot spring sites at Nakabusa hot spring located in Nagano prefecture, Japan (Wall Site [36°23′20″N 137°44′52″E], Stream Site [36°23′33″N, 137°44′52″E]). The linear distance between the two sites was approximately 170 m. Hot spring water was slightly alkaline (pH 8.5–8.9) and contained 0.046–0.138 mM of sulfide, 0.019–0.246 mM of sulfate, and 5.0–6.1 μM of ammonia (32, 33, 52). Nitrate and nitrite were not detected (32, 34). The microbial communities that developed at 72°C to 75°C were collected from the two sites (Fig. 1) on 22 July, 25 August, 18 November 2016, and 8 and 29 January 2017 using sterilized tweezers: pale tan colored microbial mats developed on a concrete wall that hot spring water runs down (Wall Site, Figs. 1A and 1B); similar pale tan colored filamentous microbial communities (streamers) were observed in a stream of the hot springs (Stream Site, Figs. 1C and 1D). The samples for DNA extraction were placed in 2.0-mL screw cap plastic tubes, frozen immediately in dry ice-ethanol slurry at the site, and stored on dry ice or at –80°C until further use. Samples for nitrogenase activity measurements were appropriately incubated *in situ* or at the laboratory without freezing (see below).

### DNA extraction and community analysis based on the 16S rRNA gene

Approximately 100 mg of samples collected on 22 July 2016 were used. Bulk DNA was isolated from samples obtained from the two different sites (Wall Site and Stream Site) using a chloroform phenol extraction protocol (55), combined with the hexadecyltrimethyl-ammonium bromide (CTAB) method. In brief, microbial cells were disrupted by bead beating with TPM buffer (50 mM Tris-HCl [pH 7.0], 1.7% [w/v] polyvinylpyrrolidone K25, 20 mM MgCl_2_), and then centrifuged. Pellets were treated as described by Noll *et al.* (55). On the other hand, supernatants were extracted using the CTAB method. This was followed by the addition of 0.95 M NaCl and 1% (w/v) CTAB and a 20-min incubation at 60°C, as well as chloroform-isoamyl alcohol and phenol-chloroform-isoamyl alcohol steps, and nucleic acids were then precipitated with isopropanol.

PCR was performed to amplify the V4 region of 16S rRNA genes using the primers 515F and 806R (9). Duplicate PCR products were pooled, purified, and quantified with the dsDNA Broad Range (BR) assay on a Qubit fluorometer (Life Technologies, Grand Island, NY, USA). PCR fragments (15 ng) were subjected to paired-end sequencing using an Illumina MiSeq platform (Illumina, San Diego, CA, USA) at Fasmac (Atsugi, Japan). Sequences were quality filtered and analyzed with the Quantitative Insights Into Microbial Ecology (QIIME) pipeline (version 1.9.0 [8]). The remaining sequences were clustered into OTUs at the 97% level and classified using the Greengenes 13-8 reference database (8, 44), as implemented in QIIME. Close relatives of representative OTUs were identified by a BLASTn analysis and phylogenetic analysis using the SILVA database (SILVA SSU Ref NR_128 database) and backbone tree (tree_SSURefNR99_ 1200slv_128) in the ARB program for a sequence analysis (43).

### Nitrogenase activity assay

Nitrogenase activity was measured using the acetylene reduction assay (2, 69). Acetylene gas generated from calcium carbide (CaC_2_+H_2_O=Ca[OH]_2_+C_2_H_2_) was used for experiments in July and August 2016, and 99.99% acetylene gas (Tatsuoka, Chiba, Japan) was used in November 2016 and January 2017. After incubations under appropriate conditions in sealed vials (see below), the production of ethylene in the gas phase was quantified using a GC-8A gas chromatograph or GC 2014 (Shimadzu, Kyoto, Japan): GC-8A was equipped with an 80/100 Porapak N (Supelco, St Louis, MO, USA) column, and GC 2014 was equipped with an 80/100 Porapak T (Supelco) column. A flame ionization detector was used with nitrogen as the carrier gas. A negative control without microbes was prepared to estimate any ethylene background generated from non-biological production.

Microbial mat samples were incubated in sealed vials containing *in situ* or artificial hot spring water and acetylene gas in the argon gas phase. Artificial hot spring water contained 1 mM NaCl, 1 mM NaH_2_PO_4_, 0.5 mM Na_2_SO_4_, 0.3 mM Na_2_S, and 1.0 mM NaHCO_3_ (pH 8.5) (40, 58). When indicated, Na_2_S, NaHCO_3_, or Na_2_SO_4_ was removed and/or 20 mM of sodium molybdenum oxide, an inhibitor of sulfate reduction, was amended (59). In the gas phase, O_2_, H_2_, and/or CO_2_ were supplied where indicated. Total protein levels in the microbial mats were measured using the DC protein assay kit (Bio-Rad Laboratories, Hercules, CA, USA) after homogenization. Homogenized mats were sonicated for 20 min, boiled to 95°C in 2% SDS for 15 min, and then centrifuged for 2 min at 19,600×*g*. The supernatant was analyzed with the Bio-Rad protein assay kit and absorbance was measured at 660 nm. A calibration was performed with bovine serum albumin standard (Bio-Rad).

### *In situ* incubation

Microbial mat samples of several grams collected from two definite points (72°C to 75°C at the Wall Site and 74°C at the Stream Site) were divided into pieces of mat (approximately 100 mg wet mass each; protein contents were measured separately), and the mats were placed into 7-mL serum vials. The vials were immediately filled with *in situ* hot spring water and sealed with butyl rubber stoppers. Argon gas (4 mL) was injected by removing a part of the hot spring water, and 0.5 mL (15% of the gas phase in July, August 2016) or 1 mL (25% of the gas phase in October 2016) of acetylene at atmosphere pressure was injected into the head space. The vials were incubated at the site: at 65°C (Wall Site) or 74°C (Stream Site) for 25 h in July, at 67°C for 18 h in August, and at 67°C for 25 h in November in 2016. Reactions were stopped by the addition of 0.5 mL of 37% formaldehyde. The vials were brought back to the laboratory on ice to assess the gas composition of the head space. Methane production from the mats collected at the Wall Site was evaluated by gas chromatography in the same measurements as those for ethylene production.

### Laboratory incubation of microbial mats

Microbial mats (7–10 g wet mass) collected on 8 January 2017 were placed into 70-mL vials with *in situ* hot spring water to a volume of 50 mL, and sealed with butyl rubber stoppers. The gas phase was air for aerobic conditions or argon gas to achieve an anaerobic pre-incubation. The vials were maintained at approximately 50°C for 10 h during transportation to the laboratory. Microbial mats were cut, three pieces of mats were combined to 450 mg wet mass, and then placed into a 20-mL vial with 8 mL of artificial hot spring water. The incubation for nitrogenase activity was performed under two different oxygen conditions in the gas phase: 0% and 5% O_2_. The vials were sealed with butyl rubber stoppers and incubated with 1.8 mL of acetylene (15% of the gas phase) at 70°C for 20 h under appropriate gas phase. The reaction was stopped by the addition of 1.5 mL of 37% formaldehyde.

### Dispersion of microbial mats

Microbial mats collected on 8 and 29 January 2017 were placed into 32-mL glass test tubes, which were immediately filled with *in situ* hot spring water and transported to the laboratory at approximately 50°C for 10 h. Cells of the microbial mats were dispersed using a glass homogenizer under flushing argon gas, washed once with washing solution (pH 8.5) containing 1 mM NaCl and 1 mM NaH_2_PO_4_, and suspended with artificial hot spring water with or without sulfide. The cell suspension was placed into a 7-mL vial with 3 mL of artificial hot spring water and incubated at 70°C for 20 h under an appropriate gas phase (Ar gas, H_2_:CO_2_ (4:1, v/v) gas, or H_2_ gas) with 0.5 mL of acetylene (15% of the gas phase) after sealing with a butyl rubber stopper. The reaction was stopped by the addition of 0.5 mL of 37% formaldehyde. A statistical analysis was performed using ANOVA followed by post-hoc Tukey-Kramer tests (α=0.05) (67), computed in R (3.4.1, https://www.R-project.org/).

### Nucleotide sequence accession numbers

16S rRNA gene sequences were deposited in DDBJ/EMBL/GenBank under BioProject accession number PRJDB6157 (PSUB007578) and BioSample accession number SAMD00089769–SAMD00089770 (SSUB008209).

## Results

### Community analysis based on 16S rRNA gene amplicon sequences

**Fig. 2:**
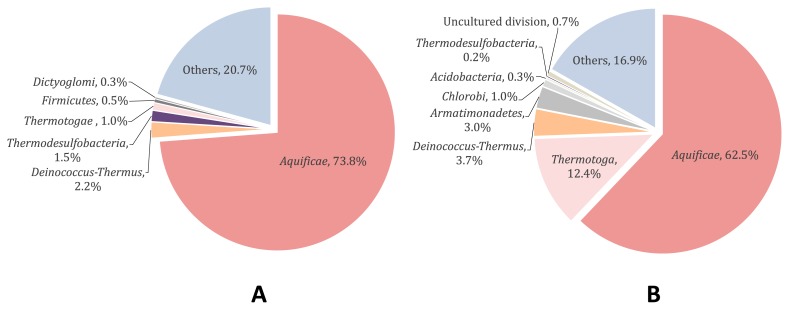
Bacterial community composition at the phylum level in microbial mats at the Wall Site (A) and streamers at the Stream Site (B) from 16S rRNA amplicon gene sequence results (V4 region). Less abundant OTUs (<0.2% read abundance for each) are shown all combined as “Others.”

Pale tan colored microbial mats and streamers developed at 72°C (Wall Site, Figs. 1A and 1B) and 75°C (Stream Site, Figs. 1C and 1D), respectively, and were collected on July 22, 2016 for a bacterial community analysis. A total of 33,049 and 38,824 paired-end 16S rRNA gene amplicon reads (V4 region) of the 16S rRNA genes were obtained from the Wall Site and Stream Site, respectively. OTUs not less than a relative abundance of 0.2% were used for the analyses shown in Fig. 2. *Aquificae* was the most predominant phylum, as previously reported for high temperature samples from Nakabusa (15, 52, 53), which accounted for 73.8% and 62.5% of all sequences at the Wall Site (Fig. 2A) and Stream Site (Fig. 2B), respectively. Sequences representing members of the two genera in the phylum *Aquificae* were predominant; one was 100% identical to those of *Sulfurihydrogenibium azorense* (65.4% and 50.3% of all sequences at the Wall Site and Stream Site, respectively) and the other was 96.0% identical to those of *Thermocrinis ruber* (5.65% and 11.3% of sequences at the Wall Site and Stream Site, respectively) (Table S1). As shown in Fig. 2 and Table S1, further sequences with not less than 0.2% relative abundance in either/both of the communities represented were 100% identical to those of *Thermus arciformis* in the phylum *Deinococcus-Thermus* (2.17% and 3.69% of all sequences at the Wall Site and Stream Site, respectively), and 98.8% identical to those of *Caldimicrobium rimae* in the phylum *Thermodesulfobacteria* (1.08% and 0.21% in Wall Site and Stream Site, respectively). Sequences representing members of the genus *Fervidobacterium* in the phylum *Thermotogae* were found in Wall Site samples only. On the other hand, sequences of *Acidobacteria*, *Armatimonadetes*, and EM3 lineage in the phylum *Thermotoga* were found in Stream Site samples.

### Nitrogenase activity measured using the acetylene reduction assay

**Fig. 3:**
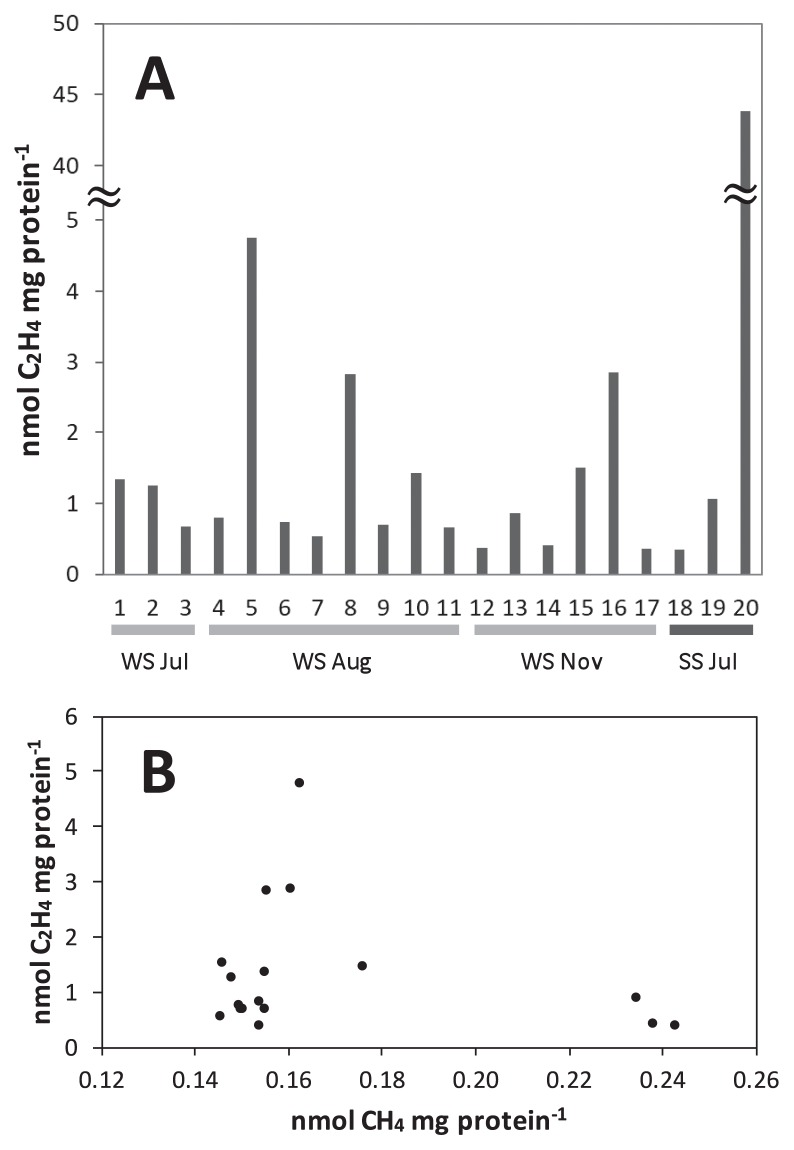
*In situ* nitrogenase activity of microbial mats and streamers **A**, Ethylene production after the *in situ* incubation of microbial mats and streamers. One to 17 were different vials containing microbial mats collected at the Wall Site (WS), and 18 to 20 were streamers collected at the Stream Site (SS). One to three and 18 to 20, four to 11, and 12 to 17 were collected on July 22, August 4, and November 8, 2016, respectively. **B**, A plot of ethylene production shown in A (1–17) versus methane production.

Collected microbial mats and streamers were incubated in hot spring water in sealed vials *in situ* at the Stream Site and Wall Site in July, August, and November 2016. As shown in Fig. 3A, ethylene production was detected and varied between 0.35 and 43.8 nmol mg protein^−1^, and also markedly varied from sample to sample. Only 4 out of 20 measurements showed activities greater than 2.5 nmol mg protein^−1^. Activities also varied in laboratory incubations: ethylene production varied between 0 and 43.8 nmol mg protein^−1^; 22 measurements out of 368 incubations from 13 different field trips showed activities of greater than 2.5 nmol mg protein^−1^ (data not shown). The results of our preliminary time course experiments that measured ethylene production at six time points in 24 h suggested that nitrogenase activity did not continue at a constant rate during the incubation period, and ethylene production was not observed in incubations that continued for more than 12 h. Therefore, we compared the total amount of ethylene in each vial instead of rates.

Methane production from the mats at the Wall Site was assessed in the same measurements as those for ethylene production and shown in the horizontal axis of Fig. 3B. Methane production varied between 0.15 and 0.24 nmol mg protein^−1^ and nitrogenase activity was plotted against methane production in this figure. Three data points of high nitrogenase activity greater than 2.5 nmol mg protein^−1^ were restricted to a methane production range between 0.16 and 0.17 nmol mg protein^−1^. Nitrogenase activity was low in the ranges of methane production both lower or higher than the range between 0.16 and 0.17 nmol mg protein^−1^. Nitrogenase activity was not affected by the addition of 2-bromo-ethane sulfonate (BES), an inhibitor of methanogenesis (data not shown) (19). Therefore, methanogens do not appear to be directly related to nitrogenase activity; however, some environmental conditions that are related to methanogenic activity may affect nitrogenase activity.

**Fig. 4:**
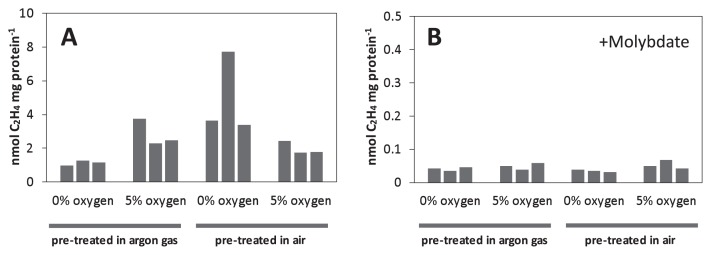
Nitrogenase activity after a pre-incubation under air or an argon atmosphere. Microbial mats were pre-incubated at approximately 50°C for 10 h under air or an argon atmosphere. After pre-cultivation, acetylene-reducing activity was measured under argon gas phase with the addition of 0% or 5% O_2_ without (**A**) or with (**B**) molybdate (20 mM). Three experimental results under each condition are shown in individual bars in a 20-mL vial. Note that the vertical scale differs between Figs. 4A and 4B.

In experiments involving the laboratory incubation of mats, we pre-treated the collected mats under different oxygen conditions (initially aerobic [air] or anaerobic conditions [argon gas]) and performed activity measurements under two different oxygen conditions: 0% or 5% oxygen. As shown in Fig. 4A, samples pre-treated in the argon gas phase showed higher ethylene production levels under 5% oxygen than under 0% oxygen conditions by a factor of ≥1.8. On the other hand, samples pre-treated under air showed ≥1.4-fold higher ethylene production levels under 0% oxygen than under 5% oxygen conditions. Since the variation in ethylene production was also large in this experiment, possibly because unavoidable inhomogeneity of the sample influenced ambient redox states during the measurements, we showed the value of each of the triple measurements instead of an average and variation.

Acetylene reduction activities were inhibited by the addition of 20 mM molybdate, an inhibitor of sulfate reduction (Fig. 4B) (25). With molybdate, air pre-treated samples showed ethylene production that was less than 1.2% and 3.9% that of molybdate-unamended samples under the 0 and 5% oxygen conditions, respectively (Figs. 4A and B). Ethylene production pre-treated in argon gas in the presence of molybdate was also less than 4.5% and 2.5% that of molybdate-unamended samples under the 0 and 5% oxygen conditions, respectively (Figs. 4A and B).

### Factors affecting nitrogen fixation in microbial mats

**Fig. 5:**
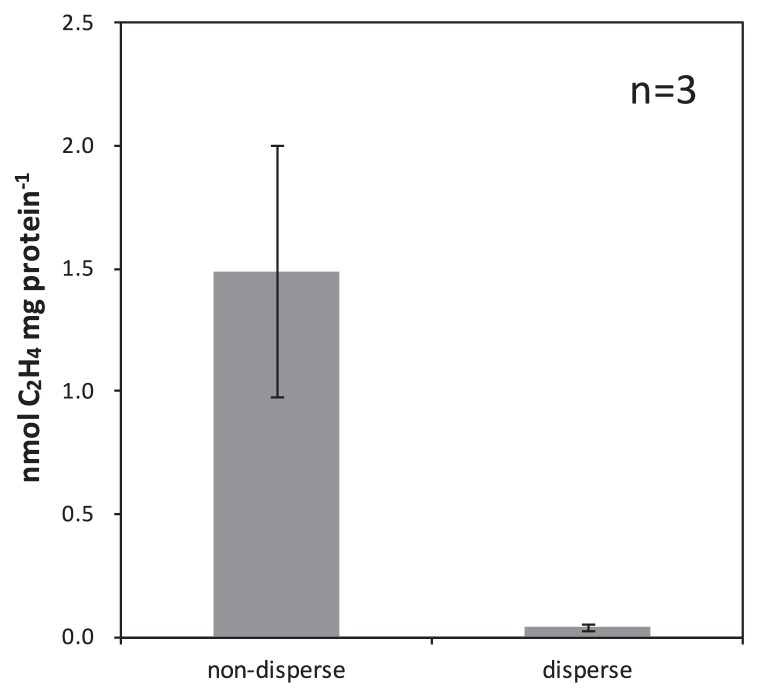
Nitrogenase activity of intact microbial mats and dispersed microbial mats Acetylene-reducing activity was measured under argon gas phase using intact mats and dispersed microbial mats. Error bars represent the standard deviation of three replicates.

Nitrogenase activity in the mats was strongly inhibited by molybdate, which is a known inhibitor of sulfate-reducing or sulfur-disproportionating metabolism, but has been reported to have no effect on nitrogenase activities (13). Nitrogenase activity in the mats may have been due to sulfate-reducing or sulfur-disproportionating metabolism and be affected by compounds related to sulfate reduction, *i.e.*, sulfate, thiosulfate, lactate, hydrogen, and carbon dioxide. In order to investigate this possibility, the effects of these compounds (sulfur sources and electron donor) were examined on nitrogenase activity measurements with mat samples, but consistent results were not obtained (data not shown). We speculated that these compounds may already be present or produced within the mats and be the cause of the inconsistent results and, thus, attempted to disperse the mats with a glass homogenizer in order to interrupt the possible cellular exchange of these compounds, *e.g.*, hydrogen, as reported by Otaki *et al.* (58). The dispersion of intact microbial mats significantly decreased nitrogenase activity; less than 4.5% of activity from that of intact microbial mats, 0.04±0.01 nmol and 1.49±0.51 nmol mg protein^−1^, respectively (Fig. 5).

**Fig. 6:**
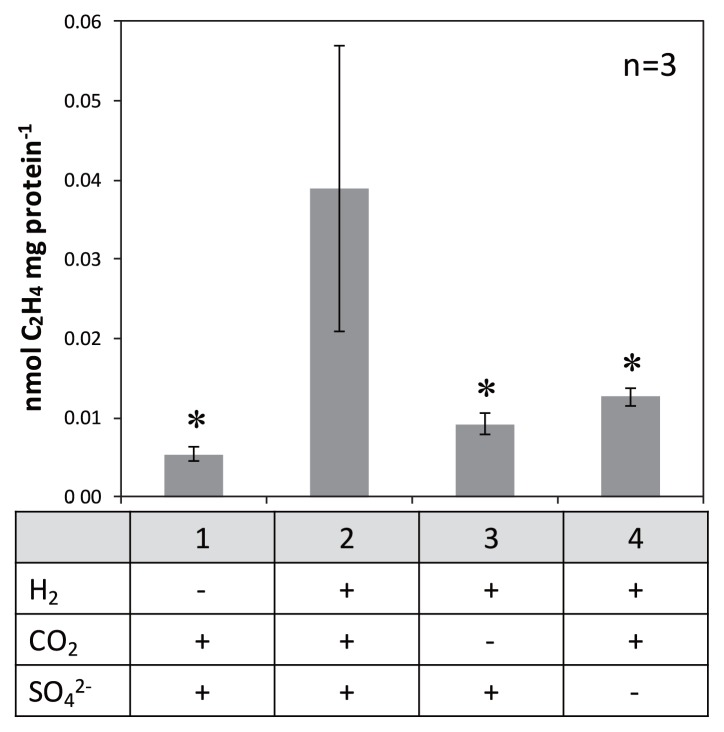
Effects of chemical conditions on nitrogenase activity of microbial mats Acetylene-reducing activity was measured using dispersed microbial mats in artificial hot spring water without Na_2_S under the following conditions: “under argon gas”, “under H_2_:CO_2_ (4:1, v/v) gas”, “under H_2_ gas”, and “under H_2_:CO_2_ (4:1, v/v) gas in artificial hot spring water containing no sodium sulfate”. Error bars represent the standard deviation of three replicates. Asterisks denote significant differences between complete (sample 2) and incomplete conditions (*P* values <0.05).

In the next experiment, the head space of the incubation vial was filled with H_2_:CO_2_ (4:1, v/v) gas, and the nitrogenase activity of dispersed microbial mats was partially recovered and approximately 7.3-fold higher (Fig. 6, sample 2, 0.039±0.018 nmol mg protein^−1^) than that without H_2_:CO_2_ (4:1, v/v) gas (Fig. 6, sample 1, 0.005±0.001 nmol mg protein^−1^) (*P*<0.05). All dispersed samples shown in Fig. 6 were incubated without sulfide to more accurately examine the effects of sulfate (*i.e.*, avoiding the possible production of sulfate by sulfide oxidation); ethylene production levels were approximately two-fold higher under the same conditions (*i.e.*, sample 1 in Fig. 6) when 0.3 mM sulfide was added (data not shown). Ethylene production was the highest in sample 2 (addition of CO_2_, H_2_ and SO_4_), but decreased to 0.009±0.001 and 0.013±0.001 nmol mg protein^−1^ when CO_2_ (Fig. 6, sample 3) or sulfate (Fig. 6, sample 4) was omitted. These results indicated that the presence of H_2_, CO_2_, and sulfate together stimulated nitrogenase activity in dispersed samples of microbial mats.

## Discussion

Nitrogen fixation activity was observed in thermophilic chemosynthetic microbial mats and streamers at temperatures higher than 70°C using acetylene reduction assays in the present study (Fig. 3). At that temperature range, the growth of anoxygenic and oxygenic photosynthetic bacteria is not supported, and only chemosynthetic and heterotrophic archaea and bacteria are present. Hamilton *et al.* (20, 21) previously reported nitrogen fixation at 86°C and pH 7.0 in sediments in Yellowstone National Park. In contrast to those findings, the present study detected nitrogenase activities in dense microbial communities (microbial mats and streamers), which have been characterized in detail in terms of community compositions (15, 53, and Fig. 2 in the present study).

*Sulfurihydrogenibium* was a dominant species of the pale tan colored dense chemosynthetic microbial communities in this and previous studies on Nakabusa hot springs as well as other alkaline sulfidic hot springs (46, 53, 73). Kimura *et al.* showed that microbial mats dominated by *Sulfurihydrogenibium* at the Stream Site in Nakabusa had a higher CO_2_ uptake rate (33) than a photosynthetic mat dominated by thermophilic cyanobacteria in Yellowstone National Park. The high productivity of *Sulfurihydrogenibium*-dominated mats suggests that the limited nitrogen sources in the spring water (5.0–6.1 μM of ammonia [32, 34] and no detectable levels of nitrate or nitrite [34, 52]) are not sufficient for the mats, and biological nitrogen fixation is needed in order to allow for productivity and a biomass to develop.

Although the chemosynthetic microbial communities at Nakabusa hot springs developed robustly in macroscopic biofilms (microbial mats) and streamers, nitrogenase activity markedly varied between different incubations. We made 13 field research trips to Nakabusa hot springs between May 2016 and March 2017 for this study and conducted repeated experiments under the same conditions; however, nitrogenase activity varied between trips and vials. A plot of nitrogenase activity against methane production suggested a possible relationship between nitrogenase and methanogenic activities, indicating a dependency on the redox state in the mats in the form of hydrogen availability (Fig. 3B). In laboratory experiments (Fig. 4), amendments with a small amount of oxygen had a positive effect on nitrogen fixation after the pre-treatment of the collected mats under the argon gas phase. On the other hand, the pre-treatment of mats under air (20% O_2_) conditions had a negative effect on the amendment with oxygen. The insides of the microbial mats were expected to be anaerobic in both pre-treatments, whereas the degree of anaerobiosis, *i.e.*, the ambient redox potential within the mats, was expected to differ between two treatments. These results indicate that in laboratory experiments with mats stored under anaerobic conditions, the ambient redox potential in mats became too negative, and the addition of oxygen changed the redox level to a slightly more oxidative state and helped to fix nitrogen under appropriate redox conditions. Methane production was not observed in laboratory incubation experiments (data not shown). Although the temperature, pH, and chemical composition of hot spring water were mostly stable at the sites, the flow rate, degree of oxygen mixing, and stage of mat development appeared to differ locally, *i.e.*, several centimeters differences may have also been a source of variability in this study. Kubo *et al.* (40) suggested that aerobic conditions are only found in the upper micrometers below the surface at 65°C, and anaerobic conditions persist in deeper layers. The chemosynthetic mats and streamers used in this study may also have contained a thin aerobic layer at the surface and anaerobic deeper layers with different ambient redox potentials, thereby enabling the co-existence of several types of metabolism. Further studies are needed in order to clarify the ambient redox potential in dense microbial mats *in situ*, as well as its relationship with cellular activity.

Nitrogen fixation in the present study was largely inhibited by molybdate, suggesting a relationship between sulfate reduction (or APS oxidation during disproportionation) and nitrogen fixation (Fig. 4B) (16). 16S rRNA gene amplicon analyses indicated the presence of bacteria of the phylum *Thermodesulfobacteria* (Fig. 2). Reported genome analyses of three species in the phylum *Thermodesulfobacteria* showed complete gene sets of nitrogenase (NC_015681, LSFI01000005, LWLG01000003); however, their nitrogen-fixing ability has not been tested. In dispersed samples of the mats, the presence of hydrogen, sulfate, and carbon dioxide was needed in order to observe apparent nitrogenase activity (Fig. 6). We added lactate, an electron donor for heterotrophic sulfate-reducing bacteria, instead of hydrogen, or thiosulfate, substances for sulfur-disproportionating bacteria, instead of sulfate; however, both results were negative for the nitrogenase assay (data not shown). These results indicate that chemoautotrophic sulfate-reducing bacteria, using hydrogen, sulfate, and carbon dioxide, are involved in nitrogen fixation. Otaki *et al.* (57) showed that hydrogen produced by fermentation was consumed by sulfate-reducing bacteria in dense microbial communities at 65°C dominated by *Chloroflexus aggregans* in Nakabusa, and a similar relationship between fermentative hydrogen producers and sulfate-reducing hydrogen consumers may be present in chemosynthetic microbial mats and streamers at temperatures higher than 70°C. The genera of *Caldicellulosiruptor* and *Fervidobacterium*, both found in Wall Site samples in the present study, have been identified as fermentative bacteria that produce hydrogen and are candidate hydrogen producers in the mats (57, 62).

The autotrophic growth of thermophilic sulfate-reducing bacteria has been reported in some lineages of bacteria, such as *Thermodesulfobacteria*, *Deltaproteobacteria*, *Desulfotomaculum* (*Firmicutes*), and *Thermodesulfobium narugense* (in a candidate phylum) (4, 22, 38, 41, 49). Among them, in the deeply branching thermophile phylum *Thermodesulfobacteria*, eight out of the 11 described species have the ability to grow autotrophically, and all species use sulfur-compound metabolism (1, 22, 25, 38, 51, 66), except for one obligatory iron-reducing species (31). We are now attempting to enrich autotrophic sulfate-reducing bacteria under diazotrophic conditions.

Hydrogeothermal environments, such as the hot springs at Nakabusa, are considered to be suitable models for studying life under the presumed conditions under which life developed. The thermophilic chemosynthetic communities examined in the present study are mainly composed of deep branched representatives of the evolutionary tree of life, and may be analogous to communities on early earth before photosynthesis evolved (Fig. 2 and Table S1) (37, 68). Although the antiquity of nitrogenase remains contentious, sedimentary N isotope records indicate a substantial history of biological nitrogen fixation on early earth (54, 70). The present results and further studies on nitrogen fixation activity in chemosynthetic communities may provide a novel insight into considerations of the ancient nitrogen cycle in microbial communities on early earth.

## Supplementary Material
