# Supplementary material for: Nitrogen Fixation in Thermophilic Chemosynthetic Microbial Communities Depending on Hydrogen, Sulfate, and Carbon Dioxide
Source: Microbes Environ. 2018 Mar 29;33(1):10–8. doi: 10.1264/jsme2.ME17134 (PMC5877335; doi:10.1264/jsme2.ME17134)
Supplement: Supplementary file 1 [file 33_10_s1.pdf]

Table S1

Relative abundance and close relatives of some OTUs (≥ 0.2% of total reads at least in one of the communities).

“0.00%” implies no reads detected. Uncultured/environmental sample sequences were included in descriptions if 16S rRNA gene sequence identity was below 90% nt to any known species.

Phylum	OTUs	Relative abundance		Descriptions (Accesion No.)	Identity (%)
		Wall Site	Steram Site		
Acidobacteria	denovo34541	0.00%	0.32%	Uncultured Acidobacteria bacterium clone YNP_SBC_BP4_B26 (HM448257)	96.4%
				Stenotrophobacter roseus strain Ac_15_C4 (NR_146022), *Stenotrophobacter namibiensis* strain Ac_17_F2 (NR_146021)	88.1%
Aquificae	denovo14971	65.4%	50.3%	*Sulfurihydrogenibium azorense* Az-Fu1 (NR_102858), *Sulfurihydrogenibium kristjanssonii* I6628 (NR_042660)	100%
	denovo48891	5.65%	11.30%	*Thermocrinis ruber* strain DSM 23557 (CP007028)	96.0%
	denovo14492	1.17%	0.37%	*Sulfurihydrogenibium azorense* Az-Fu1 (NR_102858)	96.8%
	denovo15700	0.59%	0.18%	*Hydrogenobacter subterraneus* HGP1 (NR_024729), Thermothrix azorensis strain TM (NR_104832)	99.6%
	denovo18902	0.31%	0.36%	*Thermocrinis ruber* DSM 23557 (CP007028), *Thermocrinis ruber* OC 1/4 (NR_121741)	94.1%
	denovo53671	0.23%	0.05%	*Sulfurihydrogenibium azorense* Az-Fu1 (NR_102858)	98.4%
	denovo50041	0.22%	0.07%	*Sulfurihydrogenibium azorense* Az-Fu1 (NR_102858)	98.4%
	denovo26756	0.21%	0.03%	*Sulfurihydrogenibium azorense* Az-Fu1 (NR_102858)	97.2%
	denovo29919	0.08%	0.20%	*Thermocrinis jamiesonii* GBS1 (NR_145905), Hydrogenobacter subterraneus strain HGP1 (NR_024729)	96.4%
Armatimonadetes	denovo15330	0.00%	2.48%	Uncultured Armatimonadetes bacterium, clone: ST-B09_1 (AB735180)	100%
				Pelotomaculum thermopropionicum strain SI (NR_074685)	87.3%
	denovo36368	0.00%	0.48%	Uncultured bacterium clone NKB_56_N2 (JF826973)	100%
				Carboxydothermus islandicus strain SET IS-9 (NR_108577), *Thermoterrabacterium ferrireducens* (U76364)	86.8%
Chlorobi	denovo54962	0.01%	0.98%	Uncultured Chlorobi bacterium DNA, fosmid clone: JFF027_B02 (AP011715)	99.6%
				Rhodothermus clarus (AB252420), *Rhodothermus marinus* (AY986798)	88.4%
Dictyoglomi	denovo10055	0.28%	0.11%	*Dictyoglomus turgidum* DSM 6724 (CP001251)	99.2%
Firmicutes	denovo9761	0.49%	0.01%	*Caldicellulosiruptor lactoaceticus* 6A (NR_074772), *Caldicellulosiruptor kristjanssonii* I77R1B (NR_074768), *Caldicellulosiruptor acetigenus* X6B (NR_115321)	100%
Thermodesulfobacteria	denovo20616	1.08%	0.21%	*Caldimicrobium rimae* DS (NR_044283)	98.8%
	denovo155	0.42%	0.04%	*Caldimicrobium thiodismutans* (LC055107)	100%
Thermotogae	denovo34660	1.01%	0.00%	*Fervidobacterium nodosum* Rt17-B1 (NR_074093)	100%
Thermotogae (EM3)	denovo46213	0.00%	1.15%	Uncultured Bacteroidetes bacterium clone HGM-U-2 (AB539622)	98.0%
				Actinomadura rayongensis strain RY35-68 (NR_134688)	82.7%
	denovo31056	0.01%	11.22%	Uncultured bacterium clone NKB_56_T2 (JF826975)	77.1%
				Actinomadura atramentaria strain DSM 43919 (NR_114850)	82.3%
Thermus	denovo18496	2.17%	3.69%	*Thermus arciformis* TH92 (NR_116251)	100%
uncultured division	denovo56209	0.02%	0.67%	Uncultured bacterium clone NKB_56_V 16S ribosomal RNA gene	99.6%
				Thermoanaerobacter cellulolyticus IFO 14436 (L09183)	81.7%
